# Treatment Priorities in Craniopharyngioma: Perspectives of Survivors and Caregivers

**DOI:** 10.3390/biomedicines14030664

**Published:** 2026-03-14

**Authors:** Nathalie Kayadjanian, Eugenie A. Hsu

**Affiliations:** Raymond A. Wood Foundation, 2417 Ocean Gateway, Suite B11 #108, P.O. Box 991, Ocean City, MD 21843, USA

**Keywords:** treatment priorities, craniopharyngioma, hypothalamic-pituitary brain tumors, patient registry, perspectives of caregivers and survivors

## Abstract

**Background/Objectives:** While the number and severity of comorbidities affecting survivors of craniopharyngioma (CP) are well documented, little is known about the perspectives of caregivers and survivors regarding treatment priorities. This study aimed to describe the views of caregivers and self-reported survivors on the comorbidities that most significantly impact CP survivors and to identify areas where new treatments are most needed. **Methods**: Completed surveys of 161 participants recruited in the hypothalamic–pituitary brain tumor patient registry were analyzed. **Results**: Participants represented 40% caregivers (mostly children) and 60% adult CP survivors, with notable differences in disease duration, age, CP onset, and living conditions. Seventeen health challenges were identified as most important by more than 50% of participants, including symptoms characteristic of hypothalamic dysfunction, neurological issues, and visual impairment. Notably, those differed from the most frequently experienced symptoms. No significant differences emerged between the two groups except for polydipsia, which had a greater impact on self-reported survivors. Most challenges primarily affected the survivors’ daily functioning; however, abnormal social behaviors equally impaired their ability to achieve long-term goals. Temperature dysregulation was the only symptom not deemed very or extremely important in prioritizing new treatment development. Both groups generally aligned on treatment priorities, though survivors placed a modest but significantly greater importance on fatigue and excessive daytime sleepiness, while caregivers placed a modest but significantly greater importance on obesity. **Conclusions**: Real-world survivor and caregiver perspectives on priority symptoms and treatments can inform care management, strengthen support strategies, and guide patient-focused drug development meaningful to CP survivors.

## 1. Introduction

Craniopharyngioma (CP) is a rare epithelial tumor arising along the path of the craniopharyngeal duct. CP has an incidence of 0.5 to 2 cases per million persons per year, displaying a bimodal age distribution with peak incidence rates in childhood-onset at 5 to 14 years and adult-onset at 55 to 74 years [1]. CP treatment mainly comprises neurosurgery and radiotherapy.

Despite being classified as a benign tumor by the World Health Organization, CP is associated with a high degree of morbidity due to its infiltrative tendencies and anatomical proximity to pituitary gland, hypothalamus, optic pathways, the circle of Willis, and the third ventricle. Comorbidities resulting from the tumor and/or tumor treatment include panhypopituitarism, hypothalamic dysfunction, psychosocial problems, cognitive impairment, and visual deficits [2,3]. Hypothalamic dysfunction increases the risk of behavioral problems, sleep disorders, fatigue, and excessive daytime sleepiness [4,5,6], and leads to morbid hypothalamic obesity (HO) [7]. These physical and cognitive impairments can lead to poor academic and work performance, as well as disrupted family and social relationships [8]. In addition, survivors often require lifelong medication and ongoing medical care, further impacting daily life and independence [9]. The cumulative burden of physical, cognitive, and psychosocial issues leads to a significantly reduced health-related quality-of-life [5,10,11,12,13,14], and the lowest quality of life scores of any pediatric brain tumor [15]. Survivor polysymptomatology also predicts and incurs significant caregiver burden [13].

Little is known about the views of CP survivors and caregivers on the most impactful comorbidities and priorities for new therapy development. The collection and integration of the patient- or proxy-reported patient experience, values and preferences into drug development and the regulatory decision-making process have become a priority of the Food and Drug Administration (FDA) [16], other regulatory authorities [17] and payers. Incorporating the perspectives of patients and caregivers into the drug development process can improve understanding of patient priorities, inform trial design, and support clinical trial outcome measures that translate into meaningful quality-of-life benefits. These efforts may enhance understanding of the impact of treatments on survivors’ conditions and treatment outcomes, thereby enabling more informed assessment and decision-making by regulators, health technology assessment (HTA) bodies, healthcare professionals, and patients themselves.

The study aimed to describe, for the first time, the priorities of caregivers and survivors regarding post-CP comorbidities and targets for future treatments. The objective was to obtain real-world evidence-based data on the perspectives of CP survivors and caregivers to inform the selection of symptom targets for future therapeutic development, the choice of outcome measures, and regulatory decision-making.

## 2. Materials and Methods

**Registry**

The hypothalamic–pituitary brain tumors patient registry (“registry”) was launched in May 2024. The registry is web-based and hosted on the National Organization for Rare Disorders (NORD) “IAMRARE” registry platform. The registry is compliant with US Health Information Privacy Laws, FDA regulations on electronic records, and the security requirements of the European Union General Data Protection Regulation. Registry data is only accessed by registry study personnel. All protocols, surveys, and recruitment materials have been reviewed and approved by a Central Institutional Review Board (North Star Review Board, # NB400191). The registry is governed by the Raymond A. Wood Foundation (RAWF) Registry Advisory Board members who represent stakeholders in the hypothalamic–pituitary tumors and rare disease communities, including survivors, caregivers, clinicians, and experts in registry development and governance. The registry is composed of surveys that are built in the registry in an incremental fashion to serve specific research projects and minimize the burden on participants. The present study surveys were in English and accessible worldwide.

**Participants**

Study participants were recruited via mass communications using RAWF’s channels, registry, support groups, and outreach to clinical and professional communities. Registry participants consisted of adult survivors (≥18) able to self-report and caregivers of survivors under 18 or adults unable to self-report. The study population included exclusively survivors who were diagnosed with CP and who completed all registry surveys between January and June 2025. Data from 161 participants were included in the study analyses, of which 40% (N = 64) represented caregiver-reported data and 60% (N = 97) represented self-reported data from survivors themselves.

**Ethics approval and consent to participate**

This research study was conducted in accordance with the Declaration of Helsinki, reviewed and approved in accordance with applicable US Federal regulations governing the protection of human subjects (e.g., 45 CFR part 46, and/or 21 CFR parts 50 and 56), and approved by the Central Institutional Review Board (North Star Review Board, # NB400191). All legal guardians/legally authorized representatives of CP survivors, and survivors themselves, completed an informed consent.

**Clinical Characteristics of CP Survivors**

Participants were asked to provide various information including date of birth, date of CP tumor diagnosis, HO diagnosis, and ongoing health challenges affecting CP survivors.

**Health challenges and symptoms**

To assess the ongoing post-CP sequelae symptoms and health challenges, we generated a list of 62 health issues from commonly reported symptoms [18], in addition to those that were rarely reported in the literature but frequently mentioned by caregivers and survivors in CP support groups [13]. We used the Human Phenotype Ontology that provides a standardized vocabulary to describe phenotypic abnormalities encountered in CP (https://hpo.jax.org/). The term central diabetes insipidus (DI), well known amongst the patient community, was used in this survey instead of the newly accepted denomination of arginine vasopressin deficiency (AVPD) [19].

**Symptoms of importance and treatment priorities**

Registry participants were asked to select the health challenges and symptoms that (1) had the greatest impact on CP survivors, (2) had the most negative impact on the survivor’s daily activities (e.g., not being able to work or do house chores), and (3) had (or anticipated to possibly have) the most negative impact on the survivor’s ability to achieve long-term goals as a result of CP. They were then asked to rate the level of importance for developing a new treatment for that symptom on a 5-point Likert scale (1: not important at all, 2: slightly important, 3: moderately important, 4: very important, 5: extremely important). Because of the heterogeneity of clinical manifestations of CP survivors, we expressed results in function of those who experienced them.

**Statistical Analyses**

Descriptive statistics are presented as counts (N), percentages, mean (SD), and median. Analyses comparing groups across health challenges were exploratory and did not adjust for the multiplicity of health challenges. Group differences between caregiver-reports and self-reports for the number of ongoing health challenges and the perceived importance for developing new treatments for specific health challenges were assessed using an independent Mann–Whitney test. Effect sizes were reported as a rank biserial correlation, and were categorized as trivial (<0.2), small (0.2–0.49), moderate (0.5–0.79), or large (≥0.8) [20]. Fisher’s exact test was used to compare the association between caregiver-reports and self-reports and the health challenges affecting CP survivors. A *p* value ≤ 0.050 was considered statistically significant. All statistical analyses were performed using JASP Team (2025) (version 0.95.3).

## 3. Results

### 3.1. Demographics of CP Survivors

- Caregivers reported on 64 CP survivors consisting mostly of children (N = 50, 78%) and some adults (N = 14, 22%). Self-reports represented 97 CP survivors, all of whom were adults (Table 1). Consequently, the median survivor age reported by caregivers was 13 years, compared to 25 years in self-reported survivors.
- There were more self-reported adult female than male survivors (69% versus 31% survivors). In contrast, sex distribution was more balanced in the caregiver-report group (53% females and 47% males).
- CP survivors were mostly of White ethnic background (91.25%), and residing in the United States (81%). Most survivors were single (66%), primarily living with family-of-origin when reported by caregivers (88%), or outside of family-of-origin when self-reported by adults.
- The vast majority of caregiver-reported survivors (82%) received special education or support at school, whereas the majority of self-reported adults did not (72%).

### 3.2. Clinical Characteristics of CP Survivors

#### 3.2.1. CP and HO Diagnoses

- CP diagnosis. The age at tumor diagnosis was significantly different between caregiver-reports and self-reports (U = 1129.5, *p* < 0.001) (Table 2). Most survivors reported by caregivers had childhood-onset CP. In contrast, self-reported adult survivors had a mixed childhood- and adult-onset CP.
- Disease duration. The time that has passed since CP diagnosis was significantly longer for self-reported adults compared to caregiver-reported survivors (U = 1830, *p* < 0.001) (Table 2).
- HO diagnosis. In total, 41% and 46% of survivors reported by caregivers and self received HO diagnosis, respectively, a proportion similar to what has been published in other studies [21,22]. The age of HO diagnosis was significantly different between caregiver-reports and self-reports (U = 223, *p* < 0.001) (Table 2).

#### 3.2.2. Most Frequent Health Challenges

- Caregiver-reports and self-reports reported a mean (SD) of 13.52 (6.78) and 14.14 (8.43) ongoing symptoms or health challenges affecting survivors, respectively. There were no significant differences between the two groups (U = 3.08, *p* = 0.942).
- The health challenges most frequently experienced by more than 50% survivors were hypothyroidism, fatigue, growth hormone deficiency, central adrenal insufficiency, AVPD, temperature dysregulation, obesity, and excessive daytime sleepiness, with no statistical differences between caregiver-reported and self-reported CP survivors (Table 3). In contrast, self-reported adult survivors were significantly and more frequently affected by sex hormone deficiency (70%) compared to caregiver-reported CP survivors (53%). Amongst the other health challenges and symptoms that affected more than 30% CP survivors (Table 3), decreased libido (42% versus 3%) and polydipsia (33% versus 11%) affected self-reported adults more significantly and more frequently than caregiver-reported CP survivors, respectively.

### 3.3. Most Impactful Symptoms

#### 3.3.1. Overall Impact

Seventeen health challenges were selected as symptoms with the greatest adverse impact by more than 50% of registry participants. The most impactful challenges encompassed signs and symptoms typical of hypothalamic syndrome, such as fatigue, sleep disorder, hyperphagia, obesity, temperature dysregulation, endocrine dysfunction, and emotional and behavioral problems [23]. In addition, neurological problems and visual impairment were reported as highly impactful symptoms on CP survivors (Table 4). There were no significant differences between the two groups except for polydipsia, which had a greater impact on self-reported than caregiver-reported CP survivors.

#### 3.3.2. Day-to-Day or Long-Term Impact

Most symptoms and health challenges had the most adverse impact on the survivor’s daily activities (Table 5). One exception was the decreased libido, reported by only two caregivers of CP adult survivors, for whom the long-term impact was more important. Notably, caregivers reported that abnormal social behavior, overweight, and visual impairment had an impact on both daily and anticipated long-term activities. Similarly, adult survivors self-reported an adverse impact of abnormal social behavior on both daily and long-term activities.

### 3.4. Treatment Priorities

Participants rated the development of new treatments for the most impactful symptoms affecting CP survivors as very or extremely important, except for temperature dysregulation, which was rated as only relatively or moderately important (Table 6). Caregivers and CP survivors generally agreed on treatment priorities. Of note, CP survivors assigned a modest but significantly greater priority to the development of new treatments for fatigue and excessive daytime sleepiness compared to caregivers. Conversely, the development of treatments for obesity was assigned a slightly, yet significantly, greater importance by caregivers.

## 4. Discussion

This real-world evidence study described the perspectives of adult survivors and caregivers of predominantly pediatric survivors on the most impactful comorbidities and priorities for new treatments. Seventeen symptoms or health challenges were identified as most important by more than 50% of participants. Notably, symptom importance did not mirror symptom prevalence. In addition to the symptoms associated with hypothalamic syndrome, neurological issues and visual impairment also had significant impacts on CP survivors, mostly on survivors’ daily activities. Amongst the 17 health challenges, all but temperature dysregulation were selected as top priorities for the development of new treatments. Despite differences in age, disease duration, living conditions, and role, both groups showed broad agreement, except for polydipsia, which was reported as significantly more impactful by the adult survivor group than by the caregiver group. This study highlights critical unmet needs identified by survivors and caregivers, providing direction for future treatment development in CP.

The prevalence of symptoms described by study participants is in agreement with findings reported in the literature [2,13,18,24,25], supporting the reliability of participants’ reports. The polysymptomatology affecting CP survivors is heterogeneous and depends on the tumor’s size, location, and its treatment. Accordingly, we examined which symptoms were prioritized by those who experienced them. Interestingly, we observed a clear distinction between symptom prevalence and perceived importance. Although hypothyroidism, growth hormone deficiency, and sex hormone deficiency were among the four most frequently reported conditions, they were not ranked among the top 17 symptoms of importance. In contrast, AVPD and central adrenal insufficiency (CAI) were prioritized as important. Although hypopituitary conditions are treated with hormone replacement, differences in their impact on daily life may explain why certain symptoms, but not others, were prioritized by survivors and caregivers.

Several hypotheses may explain the importance of CAI and AVPD. CAI is treated through glucocorticoid replacement. However, the life-threatening risk of adrenal crisis renders its management painstaking and lowers the quality of life [26,27,28]. The need for strict stress-dose education and emergency preparedness imposes ongoing cognitive, emotional, and behavioral demands on both patients and caregivers [28,29]. AVPD, characterized by hypotonic polyuria and polydipsia [30,31], requires lifelong AVP replacement therapy [10,32], and its management necessitates frequent, throughout-the-day engagement by patients and caregivers [33]. Hormonal replacement needs fluctuate in response to illness and lifestyle factors that alter temperature and fluid balance (e.g., heat exposure, physical activity, salt intake, stress), further complicating management and increasing the burden of care. Frequent plasma sodium monitoring, particularly in infants, young children, and survivors with adipsia, is essential to guide hydration and mitigate the risk of electrolyte imbalance (hypernatremia and hyponatremia) and their potentially life-threatening complications [10]. Future research should delineate which features of CAI and AVPD most significantly impact survivors.

Most of the 17 selected symptoms are associated with hypothalamic dysfunction, likely reflecting hypothalamic syndrome (HS), a complex clinical disorder characterizing by endocrine deficits, disordered eating, sudden weight changes, fatigue, temperature dysregulation, and a wide range of cognitive, sleep-related, and psychosocial abnormalities [7,23,34]. HS severely impacts the quality of life of both survivors [35,36] and caregivers [13,37]. Its management remains a significant challenge for clinicians [38] and presents daily difficulties for both survivors and caregivers, as highlighted in this study. Symptoms such as sleep dysregulation, fatigue, cognitive impairment, emotional lability, hyperphagia, obesity, and social impairment all pose barriers to quality of life, including the attainment and retention of relationships, educational and vocational goals, independent living, and the overall engagement with life [12,39,40,41,42,43]. Although our study did not examine the relationship between tumor characteristics, treatment, and clinical manifestations, our findings are consistent with the literature [38] and underscore the importance of addressing HS-related challenges from the perspectives of CP survivors and caregivers. The complexity of HS, along with the lack of comprehensive outcome measures that capture its multisystemic nature, likely contributes to the paucity of clinical trials beyond obesity and represents a major gap in therapeutic development for HS. The present findings should encourage future research aimed at addressing this major challenge.

In our cohort, fatigue and disordered sleep (i.e., excessive daytime sleepiness (EDS), nighttime sleep abnormalities) were identified as two of the four most important and impactful symptoms affecting daily life. While the underlying reasons for the pronounced significance attributed to these symptoms remain hypothetical, one possibility is that survivors experiencing disordered sleep may demonstrate impaired attention, reduced academic and work productivity, increased accident risk [44], and diminished cognitive capacities [44]. Although EDS and nighttime sleep abnormalities may manifest independently of fatigue, they may converge in the shared consequence of limiting the survivor’s overall engagement with life. Given the substantial day-to-day impact of fatigue and disordered sleep, future research prioritizing these issues should be considered a high priority for research and intervention.

Emotional lability and cognitive deficits emerged as the two most important symptoms for caregivers of affected survivors. Mood lability is likely highly impactful due to the management demands it poses [12,45]. By introducing persistent unpredictability, increasing behavioral management demands, disrupting family functioning, and necessitating sustained advocacy across healthcare, educational, and social systems, it is unsurprising that emotional lability is considered the most important symptom by caregivers. Cognitive impairment includes poor attention and short-term memory, and slowed processing speed and executive dysfunction [10,46,47,48], and it significantly mediates reduced adaptive functioning and independent living [49,50], possibly causing a potential lifelong caregiving burden on families [36]. Abnormal social behaviors were also included in this constellation of neuropsychological symptoms. Interestingly, social behaviors had an impact on both daily activities and the achievement of long-term goals. Although speculative, emotional lability, cognitive deficits, and abnormal social behaviors may compromise academic and vocational performance and relationships, requiring caregivers to provide extensive advocacy, coordinate accommodations and therapies, and manage the downstream consequences. Further studies should further explore how these deficits impact the perspectives of caregivers of CP survivors.

Besides reducing adaptive functioning [12], many symptoms and health challenges associated with HS result in disabilities or issues that are frequently overlooked in routine clinical practice. Sudden body changes as a result of CP like HO may alter body perception [49], lower self-esteem, and adversely affect intimacy, including sexual relationships [51]. Similarly, beyond the metabolic consequences of hyperphagia—which can lead to morbid obesity and cardiovascular comorbidities [51]—hyperphagia is accompanied by behaviors that affect all aspects of survivors’ lives [13,52,53,54] and impose a substantial burden on caregivers [13]. Children can be stigmatized because of their hyperphagia and may experience difficulties in forming friendships with their peers at school as a result of extreme food-seeking behaviors [52]. Further analyses exploring the multi-dimensional impact of these health challenges on the lives of survivors are warranted.

Our study has several limitations. First, it is cross-sectional and does not capture the trajectory of perceived treatment priorities and symptom impact over time, making causal conclusions challenging. Second, the sociodemographics of the study participants are not representative of broad ethnic groups and mostly represent persons living in the United States, limiting the generalizability of our findings. Third, our recruitment strategy may have favored participants more engaged in advocacy or survivors with more severe symptoms. Fourth, the list of health challenges and symptoms is descriptive, and further validation would be warranted. Lastly, as our study is primarily descriptive, the differences between the caregiver and survivor groups in age, disease duration, developmental stage, living conditions, and roles preclude drawing any direct causal inferences between the two groups.

Despite the disparities between the two groups, there was substantial concordance regarding the symptoms deemed important and the prioritization of treatment. Given that CP sequelae are lifelong [10,53] and that hypothalamic dysfunction is associated with worse outcomes in both pediatric [54] and adult survivors [55], the overlapping perspectives likely reflect the universality of challenges faced by all CP survivors. These findings reinforce our previous results, which demonstrated that the number and type of comorbidities are key determinants of survivors’ quality of life and caregiver burden [13]. Notably, we did not find differences in the number of health challenges affecting survivors between the two groups. The only significant difference between the two groups was for polydipsia, which adult survivors identified as an important symptom four times more often than caregivers. These findings highlight the need for further research on the impact of polydipsia in adult survivors to determine whether factors such as disease duration, living conditions, or divergent perspectives between caregivers and survivors contribute to the greater impact reported by the adult survivor group.

## 5. Conclusions

Perspectives from caregivers and adult survivors, as collected from the hypothalamic–pituitary brain tumor patient registry, provide invaluable real-world evidence regarding the lived experiences following CP. Understanding the viewpoints of those directly affected by the severity and impact of comorbidities is a critical step toward guiding the development of therapies that are truly meaningful to CP survivors and their caregivers.

## Figures and Tables

**Table 1:** Sociodemographic characteristics of registry CP survivors.

Variable	Total	Caregiver-Reports n = 64	Self-Reports
	n = 161		n = 97
Age in years, mean (SD)	27.99 (16.77)	14.67 (11.26)	36.77 (13.77)
Age in years, median (min–max)	25 (3–76)	13 (3–74)	34 (18–76)
Age category N (%)			
Infancy age 0–1	0	0	0
Toddler age 2–4	4 (2.48)	4 (6.25)	0
School-aged child age 5–12	30 (18.63)	29 (45.31)	1 (1.03)
Adolescence 13–19	31 (19.25)	22 (34.38)	9 (9.28)
Young adulthood 20–39	59 (36.65)	6 (9.38)	52 (53.61)
Middle adulthood age 40–59	31 (19.25)	2 (3.13)	30 (30.93)
Old age 60 y and above	6 (3.73)	1 (1.56)	5 (5.15)
Sex N (%)			
Female	101 (62.73)	34 (53.13)	67 (69.07)
Male	60 (37.27)	30 (46.88)	30 (30.93)
Ethnic identity, N (%)			
American Indian or Alaska Native	2 (1.24)	1 (1.56)	1 (1.03)
Native Hawaiian or Pacific Islander	1 (0.62)	0	1 (1.03)
Black or African American	10 (6.21)	1 (1.56)	9 (9.28)
Middle Eastern or North African	1 (0.62)	1 (1.56)	0
Hispanic or Latino European White	8 (4.97)	4 (6.25)	4 (4.12)
Asian	9 (5.59)	3 (4.69)	6 (6.18)
White	142 (88.20)	61 (93.75)	81 (83.51)
Other	3 (1.86)	1 (1.56)	2 (2.06)
Country of residence N (%)			
USA	131 (81.37)	54 (84.38)	77 (79.38)
Canada	11 (6.83)	5 (7.81)	6 (6.18)
UK	11 (6.83)	3 (4.69)	8 (8.25)
Columbia	1 (0.62)	0	1 (1.03)
EU	3 (1.86)	1 (1.56)	2 (2.06)
New Zealand	2 (1.24)	1 (1.56)	1 (1.03)
Australia	2 (1.24)	0	2 (2.06)
Primary living situation, N (%)			
Independent	22 (13.66)	0	22 (22.68)
With family-of-origin	84 (52.17)	56 (87.50)	28 (28.87)
With family-of-creation	35 (21.74)	5 (7.81)	30 (30.93)
With partner/boyfriend/girlfriend	7 (4.35)	0	7 (7.22)
With multi-generational family	7 (4.35)	3 (4.69)	4 (4.12)
With non-relative housemates	5 (3.11)	0	5 (5.15)
Group home/full-time support	1 (0.62)	0	1 (1.03)
Group home/part-time support	0	0	0
Other	0	0	0
Special education/support at school, N (%)			
Yes	64 (39.75)	37 (57.81)	27 (27.84)
No	97 (60.25)	27 (42.19)	70 (72.16)

**Table 2:** Clinical characteristics of CP survivors.

	Total N = 161	Caregiver-Reports N = 64	Self-Reports N = 97
Age at CP diagnosis			
Mean (SD) in years	18.5 (16.07)	10.11 (11.06)	24.03 (16.50)
Median, min–max	13, 0–76	8, 0–67	18, 3–76
**Disease duration**			
Mean (SD) in years	9.49 (10.34)	4.56 (4.43)	12.74 (11.77)
Median, min–max	6, 0–44	3, 0–18	8, 0–44
**CP onset**	N (%)	N (%)	N (%)
Childhood-onset	106 (65.84)	59 (92.19)	47 (48.45)
Adult-onset	55 (34.16)	5 (7.81)	50 (51.55)
**Age at HO diagnosis**			
Mean (SD) in years	16.25 (11.81)	9.5 (5.91)	20.16 (12.63)
Median, min–max	12, 3–58	9, 3–30	15, 5–58

**Table 3:** The most frequent symptoms/health challenges affecting CP survivors.

	Total		Caregiver-Reports		Self-Reports		Fischer’s Exact Test		
Symptoms/Health Challenges	N = 161	%	N = 64	%	N = 97	%	ODD Ratio	95% CI	*p* Value
Hypothyroidism	123	76.40	54	84.38	69	71.13	0.46	0.18–1.08	0.060
Fatigue	121	75.16	46	71.88	75	77.32	1.33	0.60–2.92	0.460
Growth hormone deficiency	110	68.32	48	75.00	62	63.92	0.59	0.27–1.25	0.167
Sex hormone deficiency	102	63.35	34	53.13	68	70.10	2.06	1.02–4.20	0.031 *
Central adrenal insufficiency	101	62.73	46	71.88	55	56.70	0.52	0.24–1.06	0.067
Central diabetes insipidus	100	62.11	43	67.19	57	58.76	0.65	0.31–1.32	0.244
Temperature dysregulation	90	55.90	35	54.69	55	56.70	1.08	0.55–2.15	0.872
Obesity	84	52.17	31	48.44	53	54.64	1.28	0.65–2.41	0.519
Excessive daytime sleepiness	84	52.17	33	51.56	51	52.58	1.04	0.53–2.06	1.00
Weight gain	74	45.96	33	51.56	41	42.27	0.69	0.35–1.36	0.262
Nighttime sleep abnormality	74	45.96	26	40.63	48	49.48	1.43	0.72–2.86	0.333
Visual impairment	69	42.86	32	50.00	37	38.14	0.62	0.31–1.23	0.147
Cognitive deficits	65	40.37	30	46.88	35	36.08	0.64	0.32–1.28	0.192
Neurological problems	62	38.51	26	40.63	36	37.11	0.86	0.43–1.74	0.741
Abnormal social behavior	60	37.27	28	43.75	32	32.99	0.64	0.31–1.28	0.185
Overweight	51	31.68	17	26.56	34	35.05	1.49	0.71–3.20	0.301
Hyperphagia/Polyphagia	49	30.43	18	28.13	31	31.96	1.20	0.57–2.57	0.727
Emotional lability	46	28.57	23	35.94	23	23.71	0.56	0.26–1.18	0.110
Decreased libido	43	26.71	2	3.13	41	42.27	22.35	5.38–198.84	<0.001 ***
Polydipsia	39	24.22	7	10.94	32	32.99	3.98	1.57–11.52	0.001 **

Participants were asked to select current and ongoing health challenges experienced by CP survivors at the time of the study. Descriptions of health challenges are indicated in Appendix A. Results are expressed as the total % of survivors reported by caregivers or themselves who experienced a given problem. Please note that this table only reports symptoms/health challenges experienced by more than 30% of the survivors (see Supplemental Table S1 for less frequent challenges). Fisher’s exact test was used to examine the association between caregiver-reports and self-reports and the presence or absence of a given symptom/health challenge. * *p* < 0.05, ** *p* < 0.01, *** *p* < 0.001.

**Table 4:** The most impactful symptoms/health challenges in CP survivors.

	Total	Caregiver-Reports	Self-Reports	Fischer’s Exact Test		
Symptoms/Health Challenges	% of Those with Symptom			ODD Ratio	95% CI	*p* Value
Fatigue	93.39	89.13	96.00	2.90	0.53–19.63	0.257
Excessive daytime sleepiness	79.76	84.85	76.47	0.58	0.14–2.04	0.415
Cognitive deficits	78.46	89.29	74.29	0.58	0.13–2.26	0.546
Neurological problems	75.81	78.26	77.78	1.28	0.33–4.85	0.767
Nighttime sleep abnormality	75.68	69.23	79.17	1.68	0.49–5.68	0.400
Obesity	72.62	70.00	75.47	1.45	0.48–4.33	0.458
Emotional lability	71.74	90.00	65.22	0.53	0.11–2.30	0.514
Hyperphagia/Polyphagia	69.39	76.47	64.52	0.70	0.15–2.87	0.754
Central adrenal insufficiency	65.35	72.09	63.64	0.85	0.34–2.09	0.834
Decreased libido	65.12	100.00	63.41	0	0–10.01	0.535
Central diabetes insipidus	65.00	77.14	64.91	1.16	0.48–2.84	0.835
Temperature dysregulation	61.11	64.71	60.00	0.89	0.34–2.30	0.828
Overweight	60.78	41.18	70.59	3.34	0.87–13.81	0.068
Abnormal social behavior	58.33	73.08	50.00	0.48	0.14–1.53	0.196
Visual impairment	57.97	54.84	62.16	1.44	0.50–4.22	0.474
Weight gain	52.70	59.38	48.78	0.71	0.25–1.94	0.490
Polydipsia	51.28	14.29	59.38	8.33	0.86–423.03	0.044 *

Participants were asked to select, among the symptoms/health challenges experienced by CP survivors, which ones impacted them the most adversely. Results are expressed as the percentage of CP survivors with a given symptom/health challenge, as reported by caregivers or survivors themselves. The denominators used for the percent calculations are indicated in Table 3. Fisher’s exact test was used to compare the association between caregiver-reports and self-reports and the symptom/health challenge of importance. * *p* < 0.05.

**Table 5:** The most impactful symptoms/health challenges on the survivor’s day-to-day life or ability to achieve long-term goals.

	Caregiver-Reports		Self-Reports	
	Day-to-Day Impact	Long-Term Impact	Day-to-Day Impact	Long-Term Impact
Symptoms/Health Challenges	% of Those with Symptom			
Fatigue	86.96	73.91	94.67	84.00
Excessive daytime sleepiness	75.76	60.61	72.55	58.82
Cognitive deficits	70.00	66.67	68.57	57.14
Neurological problems	69.23	50.00	66.67	58.33
Nighttime sleep abnormality	69.23	42.31	72.92	52.08
Obesity	67.74	54.84	69.81	62.26
Emotional lability	65.22	56.52	69.57	47.83
Hyperphagia/Polyphagia	72.22	44.44	67.74	45.16
Central adrenal insufficiency	67.39	41.30	60.00	45.45
Decreased libido	50.00	100.00	63.41	24.39
Central diabetes insipidus	65.12	37.21	61.40	28.07
Temperature dysregulation	62.86	40.00	56.36	27.27
Overweight	41.18	41.18	64.71	50.00
Abnormal social behavior	60.71	60.71	40.63	40.63
Visual impairment	46.88	46.88	62.16	48.65
Weight gain	48.48	33.33	41.46	24.39
Polydipsia	14.29	14.29	56.25	18.75

Participants were asked to select, among the symptoms/health challenges experienced by CP survivors, which ones interfered with the survivor’s daily activities (e.g., not being able to work or do house chores) and their ability to achieve long-term goals as a result of CP. Results are expressed as the percentage of CP survivors who experienced a given symptom/health challenge, as reported by caregivers (left) or survivors themselves (right). The denominators used for the calculations are indicated in Table 3. Please note that only two caregivers reported decreased libido in two survivors, thus representing 100%.

**Table 6:** Treatment priorities reported by caregivers and CP survivors.

	Treatment Priority (1–5)								
	Caregiver-Reports			Self-Reports					
Symptoms/Health Challenges	N	Mean (SD)	Median	N	Mean (SD)	Median	U	Effect Size	*p* Value
Fatigue	41	4.05 (0.87)	4	72	4.38 (0.93)	5	1101.5	0.25	0.015 *
Excessive daytime sleepiness	28	3.50 (1.14)	4	39	4.31 (0.89)	4	314	0.43	0.002 **
Cognitive deficits	25	4.12 (1.01)	4	26	4.15 (1.01)	4	321.5	0.01	0.951
Neurological problems	19	3.79 (1.18)	4	28	3.86 (1.41)	4	243.5	0.09	0.615
Nighttime sleep abnormality	18	3.67 (1.09)	4	38	4.03 (0.94)	4	276	0.19	0.226
Obesity	21	4.95 (0.22)	5	40	4.55 (0.78)	5	548	−0.31	0.010 *
Emotional lability	18	4.28 (0.83)	4.5	15	4.00 (1.00)	4	155	−0.15	0.450
Hyperphagia/Polyphagia	13	4.77 (0.44)	5	20	4.40 (0.82)	5	158	−0.22	0.224
Central adrenal insufficiency	31	4.39 (0.96)	5	35	4.06 (1.11)	4	647	−0.19	0.143
Decreased libido	2	2.50 (0.71)	2.5	26	3.62 (1.36)	4	12	0.54	0.214
Central diabetes insipidus	27	3.78 (1.31)	4	37	3.62 (1.34)	4	537.5	−0.08	0.596
Temperature dysregulation	22	2.46 (1.10)	2	33	2.67 (1.19)	3	325.5	0.10	0.310
Overweight	7	4.71 (0.76)	5	24	4.17 (1.34)	5	103.5	−0.23	0.279
Abnormal social behavior	19	4.11 (1.05)	4	16	3.81 (1.33)	4	169	−0.11	0.562
Visual impairment	17	3.94 (1.09)	4	23	4.09 (1.28)	5	172.5	0.12	0.507
Weight gain	19	4.90 (0.46)	5	20	4.70 (0.57)	5	225.5	−0.19	0.117
Polydipsia	1	N/A	5	19	3.90 (1.20)	4	N/A		

Participants were asked to select on a scale from 1 to 5 (1: not at all important, 2: slightly important, 3: moderately important, 4: very important, 5: extremely important) the level of importance for developing a new treatment for health challenges/symptoms that impacted CP survivors the most. Results are expressed as N, mean (SD), and the median amongst those experiencing a given symptom. Please note that the mean (SD) for caregiver-reported decreased libido was calculated for 2 CP survivors only. A Mann–Whitney U test was used to examine the association between caregiver-reports and self-reports and the level of importance for developing a new treatment for a given symptom/health challenge. Effect size is given by the rank biserial correlation. * *p* < 0.050, ** *p* < 0.01.

## Data Availability

Some or all datasets generated and/or analyzed during the current study are not publicly available but are available from the corresponding author on reasonable request.

## Supplementary Materials

The following supporting information can be downloaded at: https://www.mdpi.com/article/10.3390/biomedicines14030664/s1, Table S1: Current and ongoing symptoms/health challenges experienced by CP survivors.

## Appendix A

Health challenges or symptoms were presented to registry participants as follows:
Hypothyroidism (deficiency of thyroid hormone)
Fatigue (e.g., feeling tired, low energy, low motivation)
Growth hormone deficiency
Sex hormone deficiency (e.g., deficits in estrogen, testosterone, progesterone due to hypogonadism)
Central adrenal insufficiency (lack of ACTH, which leads to a decrease in the production of cortisol by the adrenal glands)
Central diabetes insipidus
Temperature dysregulation (i.e., unable to manage heat or cold, abnormal/lack of sweating or shivering)
Obesity (adults BMI ≥ 30, children ≥ 95th percentile)
Excessive daytime sleepiness (more than typical sleepiness during the day)
Weight gain
Nighttime sleep abnormality (an abnormal pattern in the quality, quantity, or characteristics of sleep, e.g., difficulties falling asleep, staying asleep, early morning wakening, sleep apnea, night terrors, sleepwalking)
Visual impairment (papilledema, slow decrease in sharpness of vision, progressive visual field defects, bitemporal hemianopia, abnormal visual field test, optic nerve atrophy)
Cognitive deficits (e.g., impairments with memory, processing, attention, learning, executive functioning)
Neurological problems (e.g., seizures, epilepsy, headaches, stroke, migraines, neuropathy, hydrocephalus)
Abnormal social behavior (e.g., social apathy, social impairment, lack of friends, social awkwardness, social withdrawal, impaired social communication, autism)
Overweight (adult BMI = 25.0–29.9, children ≥ 85th to less than 95th percentile)
Hyperphagia/Polyphagia (i.e., excessive hunger, poor satiety, food-seeking behavior)
Emotional lability (unstable emotional experiences and frequent mood changes; emotions that are easily aroused, intense, and/or disproportionate to events and circumstances)
Decreased libido (decrease in sexual desire/drive)
Polydipsia (excessive thirst, excessive drinking)
