# Supplementary material for: Treatment Priorities in Craniopharyngioma: Perspectives of Survivors and Caregivers
Source: Biomedicines. 2026 Mar 14;14(3):664. doi: 10.3390/biomedicines14030664 (PMC13024431; doi:10.3390/biomedicines14030664)
Supplement: Supplementary file 1 [file biomedicines-14-00664-s001.zip › biomedicines-4163257-supplementary.pdf]

Supplementary Table S1: Current and ongoing symptoms/health challenges experienced by CP survivors

	Total N= 161		Caregiver-reports N= 64		Self-reports N= 97	
**Symptoms**	N	%	N	%	N	%
Flat feet (pes planus)	39	24.22	14	21.88	25	25.77
Polyuria	38	23.60	11	17.19	27	27.84
Skin abnormality	37	22.98	17	26.56	20	20.62
Delayed puberty	34	21.12	11	17.19	23	23.71
Compulsive behaviors	33	20.50	15	23.44	18	18.56
Hyponatremia and/or hypernatremia	33	20.50	10	15.63	23	23.71
Hyperlipidemia	30	18.63	8	12.50	22	22.68
Short stature	28	17.39	20	31.25	8	8.25
Chronic pain	28	17.39	7	10.94	21	21.65
Growth delay	28	17.39	15	23.44	13	13.40
Behavioral acting-out	27	16.77	12	18.75	15	15.46
Nausea and vomiting	26	16.15	12	18.75	14	14.43
Nonalcoholic fatty liver disease	25	15.53	5	7.81	20	20.62
Vertigo (dizziness)	20	12.42	4	6.25	16	16.49
High blood pressure (hypertension)	18	11.18	1	1.56	17	17.53
Recurrent infections	17	10.56	7	10.94	10	10.31
Abnormality of bone mineral density	16	9.94	2	3.13	14	14.43
Allergy	16	9.94	7	10.94	9	9.28
Hearing impairment	16	9.94	5	7.81	11	11.34
Narcolepsy	14	8.70	6	9.38	8	8.25
Abnormal communication	14	8.70	6	9.38	8	8.25
Diabetes mellitus	13	8.07	2	3.13	11	11.34
Abnormality of movement	13	8.07	7	10.94	6	6.19
Other	12	7.45		0.00		0.00
Scoliosis	11	6.83	4	6.25	7	7.22
Adipsia (thirst impairment)	10	6.21	5	7.81	5	5.15
Low blood pressure (hypotension)	10	6.21	1	1.56	9	9.28
Abnormal heart rate (e.g. arrythmia)	10	6.21	3	4.69	7	7.22
Hyperprolactinemia	9	5.59	3	4.69	6	6.19
Vascular damages or disease	9	5.59	4	6.25	5	5.15
Addictions	8	4.97	1	1.56	7	7.22

Intellectual disability (IQ score below 70)	7	4.35	5	7.81	2	2.06
Bone fragility	7	4.35	2	3.13	5	5.15
Anorexia	6	3.73	1	1.56	5	5.15
Abnormal central motor function	5	3.11	2	3.13	3	3.09
Global developmental delay	5	3.11	4	6.25	1	1.03
Abnormal bone morphology	5	3.11	2	3.13	3	3.09
Underweight	3	1.86	2	3.13	1	1.03
Hyperinsulinemia	3	1.86	1	1.56	2	2.06
Psychosis	3	1.86	1	1.56	2	2.06
Precocious (early onset) puberty	2	1.24	1	1.56	1	1.03
Abnormally decreased body weight (weight loss)	1	0.62	0	0.00	1	1.03
Heart attack	1	0.62	0	0.00	1	1.03
None	1	0.62	0	0.00	1	1.03
Prefer not to answer	0	0.00	0	0.00	0	0.00

Participants were asked to select current and ongoing health challenges experienced by CP survivors at the time of the study. Results are expressed as the total % of survivors reported by caregivers or themselves who experienced a given problem. Please note that this table only reported symptoms/health challenges experienced by less than 30% of the survivors.
